# Light and Temperature as Dual Stimuli Lead to Self-Assembly of Hyperbranched Azobenzene-Terminated Poly(*N*-isopropylacrylamide)

**DOI:** 10.3390/polym8050183

**Published:** 2016-05-07

**Authors:** Wenyan Huang, Jing Yang, Yunqing Xia, Xuezi Wang, Xiaoqiang Xue, Hongjun Yang, Guifang Wang, Bibiao Jiang, Fang Li, Sridhar Komarneni

**Affiliations:** 1 Jiangsu Key Laboratory of Material Surface Science and Technology, School of Material Science and Engineering, Changzhou University, Changzhou 213164, Jiangsu, China; ellahwy@cczu.edu.cn (W.H.); 14101305@smail.cczu.edu.cn (J.Y.); 12441226@smail.cczu.edu.cn (Y.X.); 12441208@smail.cczu.edu.cn (X.W.); hjyang0519@cczu.edu.cn (H.Y.); 15101314@smail.cczu.edu.cn (F.L.); 2 Materials Research Laboratory, Materials Research Institute, The Pennsylvania State University, University Park, PA 16802, USA; wangguifang66@163.com; 3 School of Resource and Metallurgy, Guangxi University, Nanning 530004, Guangxi, China

**Keywords:** poly(*N*-isopropylacrylamide)s, azo polymers, stimuli-sensitive, self-assembly, hyperbranched

## Abstract

Hyperbranched poly(*N*-isopropylacrylamide)s (HBPNIPAMs) end-capped with different azobenzene chromophores (HBPNIPAM-Azo-OC_3_H_7_, HBPNIPAM-Azo-OCH_3_, HBPNIPAM-Azo, and HBPNIPAM-Azo-COOH) were successfully synthesized by atom transfer radical polymerization (ATRP) of *N*-isopropylacrylamide using different azobenzene-functional initiators. All HBPNIPAMs showed a similar highly branched structure, similar content of azobenzene chromophores, and similar absolute weight/average molecular weight. The different azobenzene structures at the end of the HBPNIPAMs exhibited reversible *trans-cis-trans* isomerization behavior under alternating UV and Vis irradiation, which lowered the critical solution temperature (LCST) due to different self-assembling behaviors. The spherical aggregates of HBPNIPAM-Azo-OC_3_H_7_ and HBPNIPAM-Azo-OCH_3_ containing hydrophobic para substituents either changed to bigger nanorods or increased in number, leading to a change in LCST of −2.0 and −1.0 °C, respectively, after UV irradiation. However, the unimolecular aggregates of HBPNIPAM-Azo were unchanged, while the unstable multimolecular particles of HBPNIPAM-Azo-COOH end-capped with strongly polar carboxyl groups partly dissociated to form a greater number of unimolecular aggregates and led to an LCST increase of 1.0 °C.

## 1. Introduction

Over the past few years, poly(*N*-isopropylacrylamide) (PNIPAM) [1,2] has been shown to be a typical thermo-responsive material. This material exhibits reversible hydrophilic or hydrophobic properties with a lower critical solution temperature (LCST) of around 32 °C in aqueous solution, which is quite close to the human body temperature [3]. The LCST of PNIPAM with a broad temperature range of 26–90 °C can be adjusted by the introduction of hydrophilic or hydrophobic moieties into the PNIPAM chain [4]. Therefore, PNIPAM has been used in a wide variety of areas, such as controlled drug delivery [5,6,7], smart surfaces [8,9], and smart sensors [10,11,12]. Based on its excellent performance in the above areas, the single thermo-responsive behavior of PNIPAM has been extended to dual- or multi-stimuli response by the incorporation of functional chromophore in PNIPAM. The additional stimuli, which lead to a response by tailored PNIPAM, include pH [13,14,15], ionic strength [16], light [17], *etc*.

Recently, light–temperature [17] dual stimuli-responsive polymers have received a great deal of attention in designing light-responsive materials for potential uses in remote activation, repeated reversibility, and in precisely controlling wavelength, illuminated area, direction, and intensity [18,19]. There are several reported examples of PNIPAMs or their copolymers containing light-responsive moieties [17,19], such as azobenzene, fulgimide, spiropyran, and dithienylethene groups. Azobenzene-containing PNIPAMs, in particular, have received considerable attention. The unique reversible *trans-cis-trans* isomerization cycles of the azobenzene chromophore can be smoothly completed by alternating UV and visible light irradiation, which lead to mesoscopic to large-scale shape changes and movement of the dipole moment of the rigid azobenzene chromophore. The macroscopic properties could be triggered by light, resulting in an LCST increase followed by significant changes in the polarity of PNIPAM chains [20,21,22]. Feng *et al.* [23] reported that both temperature and light irradiation could induce a reversible change of hydrophobicity of the micellar cores in amphiphilic diblock copolymers composed of ethylene oxide, azobenzene-containing methacrylate and NIPAM units.

Moreover, azobenzene-terminated PNIPAMs can also be used for dual light- and temperature-responsive material. Akiyama and Tamaoki [24] firstly reported that the LCST shifts of PNIPAM with a single photoresponsive azobenzene unit occurred after exposure to UV and visible light, and these LCST shifts depended strongly on the amount of azobenzene in polymers [25]. Hyperbranched structure also effected different LCST changes in PNIPAM terminating with an azobenzene group. When azobenzene-terminated polymers were designed with a unique amphiphilic azobenzene-containing hyperbranched poly(ether amine) (hPEA-AZO) [26], the tunable cloud point (CP) unusually downshifted during UV irradiation. The more regular *trans*- form of azobenzene changed into the *cis*- form after UV irradiation, and the *cis*- form could not pack as closely as *trans*- form in the core of hPEA211-AZO nanoparticles, resulting in a larger size of hPEA-AZO nanoparticles. Zhang *et al.* [27] investigated hyperbranched poly(ethylenimine) terminated with the azobenzene chromophores, and the results showed that UV irradiation increased the CP value at pH ≈ 7, while the opposite occurred at pH ≈ 9. These results indicated that the changes in the LCST were affected by the topology of the polymer. However, the effect of azobenzene structure on the self-assembly behavior of hyperbranched PNIPAM and LCST under light irradiation has not yet been investigated in detail. In this paper, hyperbranched PNIPAMs end-capped with different azobenzene groups were successfully synthesized by atom transfer radical polymerization (ATRP) in order to investigate the light–temperature dual stimuli-induced behavior of the differently modified polymers in detail.

## 2. Experimental Section

### 2.1. Materials

*p*-Anisidine, *p*-aminobenzoic acid, phenol, aniline, *N*,*N*′-methylenebis(acrylamide) (MBA; Analytical reagent; Shanghai Chemical Reagent Co. Ltd., Shanghai, China), 2-bromoisobutyryl bromide (98%; Sigma-Aldrich, St. Louis, MO, USA), 4-propoxyaniline (98%; Sigma-Aldrich), and *tert*-Butyl α-bromo*iso*butyrate (*t*-BB*i*B, 98%; Aldrich) were used as received. *N*-Isopropylacrylamide (NIPAM; 98%; Sigma-Aldrich) was purified three times by recrystallization from cyclohexane. Copper (I) chloride (CuCl; Chemical pure; Shanghai Chemical Reagent Co. Ltd.) was purified by washing with acetic acid and acetone, and then dried *in vacuo*. Tris[2-(dimethylamino)ethyl]amine (Me_6_TREN) was synthesized according to the previously described procedure in the literature (^1^H-NMR spectrum is provided in the Supplementary Materials, Figure S1) [28]. Other reagents were purified using the standard procedures before use.

### 2.2. Analysis and Characterization

The purities of products were determined using a Waters e2695 high performance liquid chromatography unit (HPLC, Waters, Milford, MA, USA), comprising an XBridge C18 column (5 μm, 4.6 mm × 250 mm) and a Waters 2998 UV detector (Waters, Milford, MA, USA). A mixture of acetonitrile (HPLC grade) and water (deionized and filtered with 0.45 μm membrane filter) at the gradient volume ratio (40/60~90/10~40/60) was used as the eluent. The flow rate was at 1.0 mL·min^−1^ and the column temperature was 30 °C. Conversion of the reactants was determined using an HP-689 gas chromatography unit (GC, Kexiao, Shanghai, China) equipped with an HP-5 column (30 m × 0.54 mm × 0.5 μm). Isopropyl alcohol was used as the internal standard. The carrier gas was hydrogen and the flow rate was set at 1 mL·min^−1^. The column temperature was increased from a starting value of 80 °C at sample injection to a maximum of 230 °C at the rate of 10 °C·min^−1^. The peaks were identified using chromatograms of the corresponding pure reactants. The number-average molecular weight (*M*_n GPC_s) and molecular weight distributions (*M*_w_/*M*_n_s) of the polymers were determined with a Waters 1515 gel permeation chromatography (GPC, Waters, Milford, MA, USA) device equipped with a refractive index detector, a Wyatt DAWN HELEOS II light scattering photometer (Wyatt, Santa Barbara, CA, USA), and a Wyatt ViscoStar viscometer (Wyatt, Santa Barbara, CA, USA), using HR1, HR3, and HR4 columns with molecular weights in the range of 100–500,000 g·mol^−1^, which were calibrated with polystyrene (PS) standard samples. THF was used as the eluent at a flow rate of 1.0 mL·min^−1^, operating at 30 °C. ^1^H-NMR spectra of the compounds and polymers were recorded on a Bruker ARX-500 type nuclear magnetic resonance instrument (Bruker, San Antonio, TX, USA), using CDCl_3_ as solvent and tetramethyl-silane (TMS) as the internal standard. Ultraviolet visible (UV–Vis) absorption spectra and the transmittance at 550 nm light for lower critical solution temperature (LCST) of polymer solution (2 mg·mL^−1^) in water were performed on an Agilent Cary 100 equipped with a Cary dual cell Peltier accessory (Agilent, Santa Clara, CA, USA). Dynamic light scattering (DLS) data were acquired using an ALV/CGS-3 compact goniometer system (ALV, Hammaburg, Germany) at 25 °C, and am He–Ne laser operating at a wavelength of λ_0_ = 632.8 nm was used as a light source. Sample aqueous solutions for analysis (2 mg·mL^−1^) were poured into the sample bottle, which was placed in a sample cell filled with toluene used as the immersion liquid. Temperature control of the sample was provided by an external thermostated circulating bath. The accessible scattering angles range from 30° to 150°. Transmission electron microscopy (TEM) was done using a JEM-2100 TEM (JEOL, Tokyo, Japan) with a 100 kV accelerating voltage.

### 2.3. Synthesis of 4-Propoxy-4′-Hydroxyazobenzene (C_3_H_7_O-Azo-OH), 4-Methoxy-4′-Hydroxyazobenzene (CH_3_O-Azo-OH), 4-Hydroxyazobenzene (Azo-OH), and 4-Carboxyl-4′-Hydroxyazobenzene (HOOC-Azo-OH)

The above four compounds could be synthesized using a similar method. For example, the following general procedure was used for the synthesis of CH_3_O-Azo-OH: Firstly, *p*-anisidine (2.44 g, 20.0 mmol) was dissolved in concentrated HCl (6 mL) and deionized water (24 mL) in a 50 mL three-necked flask, and cooled to 0–5 °C in an ice bath. Then aqueous solution (10 mL) of NaNO_2_ (1.24 g, 18.0 mmol) was added slowly to the above mixture, and a yellow transparent diazonium salt solution was obtained by stirring at 0–5 °C for 30 min. A coupling solution of phenol (1.88 g, 20.0 mmol), Na_2_CO_3_ (3.18 g, 30.0 mmol), NaHCO_3_ (2.52 g, 30.0 mmol), and deionized water (50 mL) was also prepared and cooled to 0 °C. Then diazonium salt solution was added dropwise to the coupling solution. During this period of steady reaction, the pH of the mixture was adjusted in the range of 8–9 using a 40% NaOH solution, and finally the mixture was further stirred for 5 h at 0–5 °C. A red-orange precipitate was collected by filtration, washed with deionized water several times, and dried under a vacuum. The crude products were recrystallized three times from ethanol to achieve pure CH_3_O-Azo-OH as a red-orange crystal (3.87 g, yield: 85.2%). The purity was 97.8% (HPLC); ^1^H-NMR (400MHz, CDCl_3_), δ (TMS, ppm): 7.99–7.85 (m, 4H, ArH), 7.37–7.19 (d, 2H, ArH), 7.09–6.92 (d, 2H, ArH), 3.91 (s, 3H, CH_3_O). C_3_H_7_O-Azo-OH, Azo-OH, and HOOC-Azo-OH crystals were prepared using 4-propoxyaniline, aniline, *p*-aminobenzoic acid and phenol in the same manner as that of the CH_3_O-Azo-OH and their characteristics are given below.

C_3_H_7_O-Azo-OH: The purity was 95.8% (HPLC); ^1^H-NMR (400 MHz, CDCl_3_), δ (TMS, ppm): 7.76–7.89 (m, 4H, ArH), 6.96–7.01 (d, 2H, ArH), 6.88–6.95 (d, 2H, ArH), 3.96–4.04 (t, 2H, CH_2_O), 1.77–1.91 (m, 2H, CH_2_), 1.00–1.10 (t, 3H, CH_3_).

Azo-OH: The purity was 98.1% (HPLC); ^1^H-NMR (400 MHz, CDCl_3_), δ (TMS, ppm): 8.08–7.76 (m, 4H, ArH), 7.68–7.44 (d, 3H, ArH), 7.43–7.25 (d, 2H, ArH).

HOOC-Azo-OH: The purity was 96.1% (HPLC); ^1^H-NMR (400 MHz, CDCl_3_), δ (TMS, ppm): 8.31–8.18 (m, 2H, ArH), 8.09–7.92 (d, 4H, ArH), 7.43–7.23 (d, 2H, ArH).

### 2.4. Synthesis of 4-Propoxy-4′-(2-Bromopropionyloxy)azobenzene (C_3_H_7_O-Azo-Br), 4-Methoxy-4′-(2-Bromopropionyloxy)azobenzene (CH_3_O-Azo-Br), 4-(2-Bromopropionyloxy)azobenzene (Azo-Br), and 4-Carboxyl-4′-(2-Bromopropionyloxy)azobenzene (HOOC-Azo-Br)

The above four compounds could be synthesized using a similar method. As an example, the following general procedure was used for the preparation of CH_3_O-Azo-Br: CH_3_O-Azo-OH (2.28 g, 10.0 mmol), freshly distilled THF (50 mL), and freshly distilled triethylamine (1.22 g, 12.0 mmol) were added to a 250-mL three-necked flask. The solution was cooled to 0–5 °C, and stirred in an ice bath. 2-Bromoisobutyryl bromide (1.48 mL, 12.0 mmol) was diluted in dry THF (20 mL), and the solution was added dropwise to the above cooled and stirred mixture. The reaction mixture was vigorously stirred for 5 h at 0–5 °C and then kept at room temperature overnight. The mixture was filtered, and the filtrate was evaporated under a vacuum. The remaining yellow mixture was dissolved in ethyl acetate (50 mL) and washed with 5% Na_2_CO_3_ aqueous solution and deionized water three times, dried with anhydrous MgSO_4_ overnight, filtered, and evaporated under reduced pressure. The final crude product was recrystallized three times from ethanol to yield a yellow solid (2.49 g, yield: 66.1%). The purity was 98.2% (HPLC); ^1^H-NMR (400 MHz, CDCl_3_), δ (TMS, ppm): 8.00–7.84 (m, 4H, ArH), 7.38–7.18 (d, 2H, ArH), 7.09–6.92 (d, 2H, ArH), 3.92 (s, 3H, CH_3_O), 2.10 (s, 6H, CH_3_C) (^1^H-NMR spectrum is provided in the Supplementary Materials, Figure S2). C_3_H_7_O-Azo-Br, Azo-Br, and HOOC-Azo-Br crystals were prepared using C_3_H_7_O-Azo-OH, Azo-OH, and HOOC-Azo-OH in the same manner as that of the CH_3_O-Azo-Br and their characteristics are given below.

C_3_H_7_O-Azo-Br: The purity was 98.3% (HPLC); ^1^H-NMR (400 MHz, CDCl_3_), δ (TMS, ppm): 7.85–8.00 (m, 4H, ArH), 7.22–7.32 (d, 2H, ArH), 6.97–7.05 (d, 2H, ArH), 3.96–4.06 (t, 2H, CH_2_O), 2.10 (s, 6H, C(CH_3_)_2_), 1.79–1.91 (m, 2H, CH_2_), 1.02–1.11 (t, 3H, CH_3_) (^1^H-NMR spectrum is provided in Supplementary Materials, Figure S3).

Azo-Br: The purity was 96.8% (HPLC); ^1^H-NMR (400 MHz, CDCl_3_), δ (TMS, ppm): 8.09–7.74 (m, 4H, ArH), 7.69–7.44 (d, 3H, ArH), 7.44–7.25 (d, 2H, ArH), 2.04 (s, 6H, CH_3_C) (^1^H-NMR spectrum is provided in Supplementary Materials, Figure S4).

HOOC-Azo-Br: The purity was 97.5% (HPLC); ^1^H-NMR (400 MHz, CDCl_3_), δ (TMS, ppm): 8.32–8.18 (m, 2H, ArH), 8.09–7.91 (d, 4H, ArH), 7.44–7.21 (d, 2H, ArH), 2.10 (s, 6H, CH_3_C) (^1^H-NMR spectrum is provided in Supplementary Materials, Figure S5).

### 2.5. Synthesis of Hyperbranched Poly(N-isopropylacrylamide)s (HBPNIPAM-Azo-OC_3_H_7_, HBPNIPAM-Azo-OCH_3_, HBPNIPAM-Azo, and HBPNIPAM-Azo-COOH) and Linear Poly(N-isopropylacrylamide) (LPNIPAM)

The general procedure was used as follows: Me_6_TREN (0.230 g, 1.00 mmol), CuCl (0.033 g, 0.33 mmol), isopropyl alcohol (3.00 g) and deionized water (1.00 g) were added to a 25-mL Schlenk flask equipped with a stirbar. The flask was cycled three times between a vacuum under an ice bath and argon at room temperature, and then the solution was stirred in argon for 30 min. C_3_H_7_O-AZO-Br (0.135 g, 0.33 mmol), MBA (0.046 g, 0.30 mmol), and NIPAM (1.13 g, 10.00 mmol) were added to the reaction vessel. After three freeze–pump–thaw cycles, the flask was sealed and placed in an oil bath to start the polymerization at 25 °C. After a preset reaction time, the polymerization was stopped by adding CuBr_2_ (0.074 g, 0.33 mmol). After solvent evaporation under reduced pressure, the crude polymers were dissolved in about 15 mL THF, passed through a small neutral Al_2_O_3_ chromatographic column to remove the Cu^2+^ deactivator, and precipitated by dropwise addition to cold hexane (200 mL). The precipitates were filtered, redissolved in THF, isolated by precipitation into hexane, and dried to a constant weight at room temperature in a vacuum. Thus, pure HBPNIPAM-Azo-OC_3_H_7_ was obtained. The other hyperbranched polymers, HBPNIPAM-Azo-OCH_3_, HBPNIPAM-Azo, and HBPNIPAM-Azo-COOH, were prepared using a similar procedure but with CH_3_O-Azo-Br, Azo-Br and HOOC-Azo-Br, respectively, as initiators, and LPNIPAM was also obtained using a similar procedure with a predetermined molar ratio ([NIPAM]_0_:[*t*-BB*i*B]_0_:[CuCl]_0_:[Me_6_TREN]_0_ = 30:1:1:3) using *t*-BB*i*B as initiator. The obtained polymers were dissolved in THF and filtered by PTFE film for GPC analysis. The samples were dissolved in CDCl_3_, and measured by ^1^H-NMR spectroscopy. The polymers were dissolved in distilled water, and the HBPNIPAMs (2 mg·mL^−1^) solutions were analyzed by TEM, DLS, and UV–Vis absorption tests.

## 3. Results and Discussion

### 3.1. Preparation of Hyperbranched Poly(N-isopropylacrylamide)s End-Capped with Different Azobenzene Chromophores (HBPNIPAM-Azo-OC_3_H_7_, HBPNIPAM-Azo-OCH_3_, HBPNIPAM-Azo, and HBPNIPAM-Azo-COOH)

**Scheme 1:**
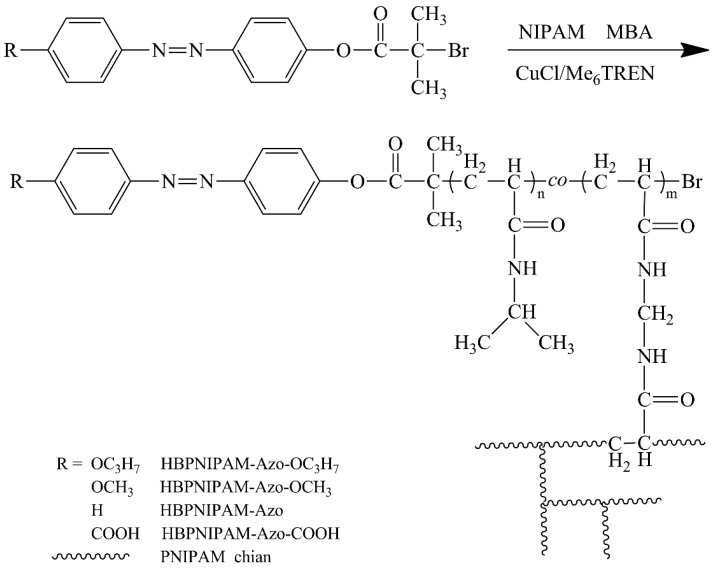
Synthetic route of the azobenzene-terminated hyperbranched poly(*N*-isopropylacrylamide)s (HBPNIPAMs).

**Figure 1:**
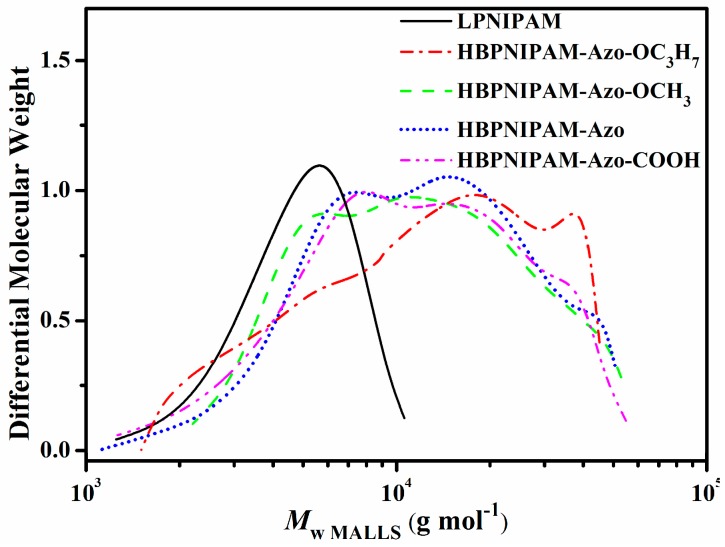
The differential molecular weight distribution curves of the linear poly(*N*-isopropylacrylamide) (LPNIPAM, *M*_n GPC_ = 4100 g·mol^−1^, *M*_w_/*M*_n_ = 1.23) and hyperbranched PNIPAMs (HBPNIPAM-Azo-OC_3_H_7_: *M*_n GPC_ = 7300 g·mol^−1^, *M*_w_/*M*_n_ = 2.13; HBPNIPAM-Azo-OCH_3_: *M*_n GPC_ = 8400 g·mol^−1^, *M*_w_/*M*_n_ = 1.82; HBPNIPAM-Azo: *M*_n GPC_ = 8800 g·mol^−1^, *M*_w_/*M*_n_ = 1.78; HBPNIPAM-Azo-COOH: *M*_n GPC_ = 7400 g·mol^−1^, *M*_w_/*M*_n_ = 2.05).

Hyperbranched polymers were previously prepared through controlled/“living” radical polymerization in the presence of branching agents [29,30,31,32,33,34,35,36,37,38,39,40,41,42,43,44,45] and hence it was hypothesized that the hyperbranched poly(*N*-isopropylacrylamide) (PNIPAM) could also be synthesized by atom transfer radical polymerization (ATRP) of PNIPAM and *N*,*N*′-methylenebis(acrylamide) (MBA) [46,47,48]. Therefore, the ATRP of NIPAM using different azobenzene-functional initiators was carried out using Scheme 1 under the condition of isopropyl alcohol:water = 2:1 (m/m), *T* = 25 °C, and [NIPAM]_0_:[MBA]_0_:[initiator]_0_:[CuCl]_0_:[Me_6_TREN]_0_ = 30:0.9:1:1:3. The results (Table 1) show that all the conversions of the NIPAM were above 90%, and the molecular weights of HBPNIPAM-Azo-OC_3_H_7_, HBPNIPAM-Azo-OCH_3_, HBPNIPAM-Azo, and HBPNIPAM-Azo-COOH were determined to be 7300, 8400, 8800, and 7400 g·mol^−1^, respectively. The above molecular weights of HBPNIPAMs were much higher than that of linear PNIPAM (LPNIPAM, *M*_n GPC_ = 4100 g·mol^−1^). The newly synthesized HBPNIPAMs showed relatively broad molecular weight distributions (*M*_w_/*M*_n_ > 1.78), which confirmed their highly branched structures. In addition, Figure 1 shows the differential molecular weight distribution curves of the HBPNIPAMs. Compared with the single peak of normal distribution for LPNIPAM, the curves of HBPNIPAMs showed multiple peaks, which suggested the existence of three components from the slightly branched chains to the highly branched chains [40,41,42,43,44,45]. All the HBPNIPAMs with similar distribution curves also suggested that almost the same branched chains could be obtained using a similar functional initiator. Due to the branched structures [40,41,42,43,44,45], the absolute weight/average molecular weights of HBPNIPAMs (*M*_w MALLS_) were determined using GPC equipped with the light scattering photometer, and these values were found to be above 50,000 g/mol (Table 1), which suggested that there were more than 12 linear chains in the HBPNIPAMs (*n* = *M*_w MALLS_/(*M*_LPNIPAM_ × *M*_w_/*M*_n_)).

**Figure 2:**
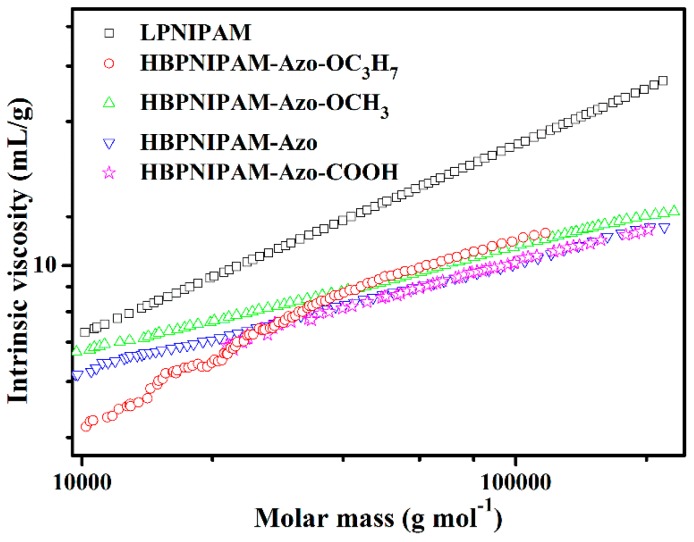
The Mark–Houwink plots for the linear poly(*N*-isopropylacrylamide) (LPNIPAM, *M*_n GPC_ = 4100 g·mol^−1^, *M*_w_/*M*_n_ = 1.23) and hyperbranched PNIPAMs (HBPNIPAM-Azo-OC_3_H_7_: *M*_n GPC_ = 7300 g·mol^−1^, *M*_w_/*M*_n_ = 2.13; HBPNIPAM-Azo-OCH_3_: *M*_n GPC_ = 8400 g·mol^−1^, *M*_w_/*M*_n_ = 1.82; HBPNIPAM-Azo: *M*_n GPC_ = 8800 g·mol^−1^, *M*_w_/*M*_n_ = 1.78; HBPNIPAM-Azo-COOH: *M*_n GPC_ = 7400 g·mol^−1^, *M*_w_/*M*_n_ = 2.05).

**Figure 3:**
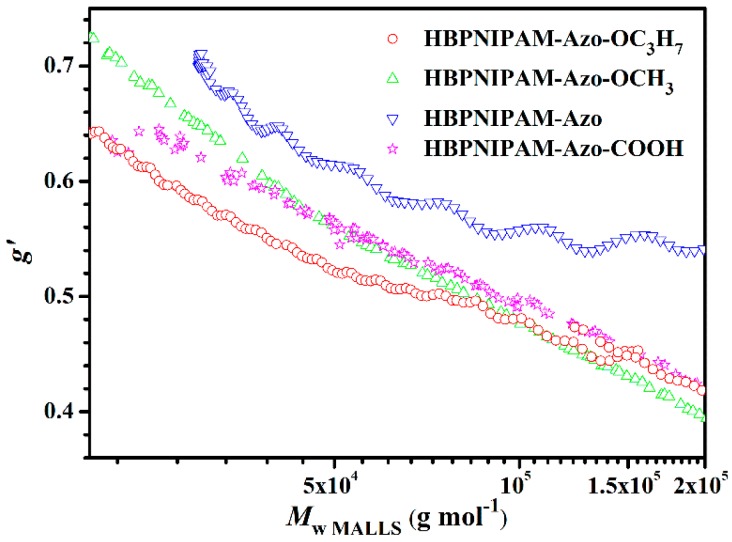
Variation of the Zimm branching factor (*g*′) with molecular weight for hyperbranched poly(*N*-isopropylacrylamide)s (HBPNIPAM-Azo-OC_3_H_7_: *M*_n GPC_ = 7300 g·mol^−1^, *M*_w_/*M*_n_ = 2.13; HBPNIPAM-Azo-OCH_3_: *M*_n GPC_ = 8400 g·mol^−1^, *M*_w_/*M*_n_ = 1.82; HBPNIPAM-Azo: *M*_n GPC_ = 8800 g·mol^−1^, *M*_w_/*M*_n_ = 1.78; HBPNIPAM-Azo-COOH: *M*_n GPC_ = 7400 g·mol^−1^, *M*_w_/*M*_n_ = 2.05).

The degree of branching of the HBPNIPAMs could also be determined by measuring viscosity with GPC equipped with an intrinsic viscosity (IV) measuring attachment. Figure 2 shows the Mark–Houwink plots of molecular weight for the HBPNPAMs and LPNIPAM. According to the Mark–Houwink equation ([η] = *KM*^α^), the Mark–Houwink exponent, α, was the slope of the curve in Figure 2, which was closely tied to the degree of branching. The smaller α indicated a higher degree of branching. It is clear from Figure 2 that the α values of the HBPNIPAMs were much lower than that of LPNIPAM; all the α values are listed in Table 1. The HBPNIPAMs showed more or less similar curves leading to almost the same degree of branching. Moreover, the Zimm branching factor, *g*′ (*g*′ = IV_branched_/IV_linear_) [49], is typically used as a qualitative indicator of the degree of branching. It is clear that the *g*′ value of the LPNIPAM is 1 (Table 1) while the HBPNIPAMs with higher degree of branching showed smaller *g*′ values, *i.e.*, below 1. As shown in Figure 3, the *g*′ value of all the HBPNIPAMs decreased gradually with increasing molecular weight, suggesting a higher branched structure with the higher molecular weight. Moreover, all the curves for the HBPNIPAMs showed a similar decreasing trend and showed similar average values of *g*′, *i.e.*, 0.60, 0.73, 0.65, and 0.59 (Table 1). Thus a similar degree of branching of the HBPNIPAMs with the same molecular weight was obtained using different functional initiators under the same polymerization system.

**Figure 4:**
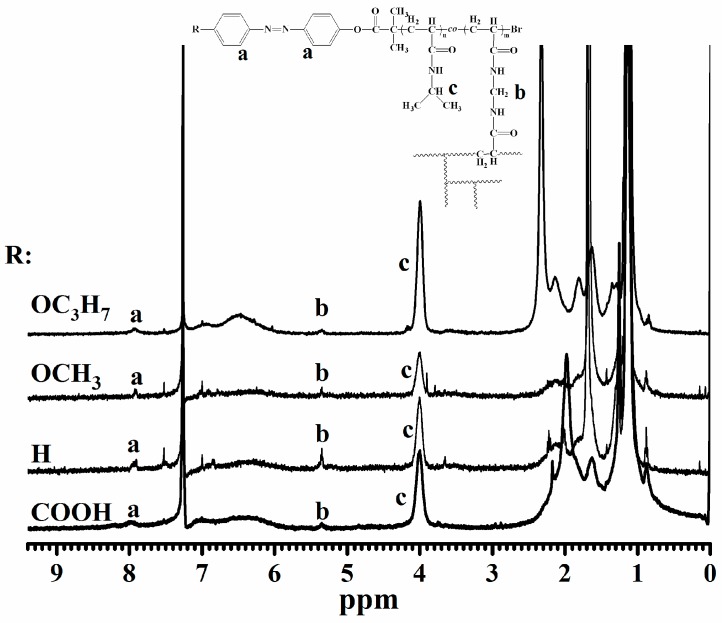
^1^H-NMR spectra of the hyperbranched poly(*N*-isopropylacrylamide)s (HBPNIPAM-Azo-OC_3_H_7_: *M*_n GPC_ = 7300 g·mol^−1^, *M*_w_/*M*_n_ = 2.13; HBPNIPAM-Azo-OCH_3_: *M*_n GPC_ = 8400 g·mol^−1^, *M*_w_/*M*_n_ = 1.82; HBPNIPAM-Azo: *M*_n GPC_ = 8800 g·mol^−1^, *M*_w_/*M*_n_ = 1.78; HBPNIPAM-Azo-COOH: *M*_n GPC_ = 7400 g·mol^−1^, *M*_w_/*M*_n_ = 2.05) in CDCl_3_.

To confirm the HBPNIPAM structures with the end azobenzene group, the obtained polymers were characterized by ^1^H-NMR spectra (Figure 4). The strong signals at 3.8–4.2 ppm (labeled as “c” in Figure 4) were assigned to the methenyl protons of the NIPAM units, and the multiple signals in the range of approximately 0.7–2.6 ppm were assigned to other alkyl protons of the NIPAM units. The characteristic signals at 3.8–4.2 ppm confirmed the existence of the NIPAM units. The weak signals at 5.3 ppm (labeled as “b“ in Figure 4) corresponded to protons in the methylene from MBA, which indicated that MBA units entered the polymer chain, forming branched structures. Moreover, the weak signals attributed to the phenyl protons of the azobenzene at the end of PNIPAMs were observed around 7.8–8.1 ppm (labeled as “a“ in Figure 4), which confirmed the existence of the azobenzene moiety at the end of the polymer chains. The content (n) of azobenzene in the PNIPAMs could be estimated based on the integrals calculated by ^1^H-NMR spectra, as given by the following equation:

(1) $$n=\frac{I_{a}/4}{I_{a}/4+I_{b}/2+I_{c}}\times 100\%$$

where, *I*_a_, *I*_b_, and *I*_c_ are the integrals of the signals at 7.8–8.1, 5.3, and 3.8–4.2 ppm, respectively (Figure 4). The *n* values of HBPNIPAM-Azo-OC_3_H_7_, HBPNIPAM-Azo-OCH_3_, HBPNIPAM-Azo, and HBPNIPAM-Azo-COOH were calculated to be 0.031, 0.030, 0.029, and 0.031, respectively. The above results showed that the contents of azobenzene were consistent with the corresponding theoretical values (about 0.032), which confirmed that all the HBPNIPAMs were end-capped with different azobenzene chromophores but with about the same contents. According to the integration areas of signals b and c, the ratio of NIPAM/MBA in the HBPNIPAMs (2*I*_c_/*I*_b_) was also calculated to be about 31, which was close to theoretical values (30/0.9 = 33).

### 3.2. Photoisomerization Behavior of Hyperbranched Poly(N-isopropylacrylamide)s End-Capped with Different Azobenzene Chromophores (HBPNIPAM-Azo-OC_3_H_7_, HBPNIPAM-Azo-OCH_3_, HBPNIPAM-Azo, and HBPNIPAM-Azo-COOH)

**Figure 5:**
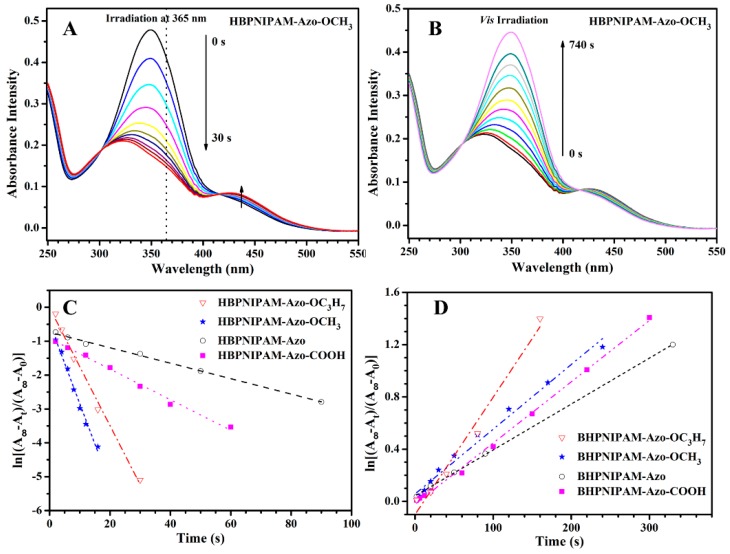
Evolution of UV–Vis absorption spectra of HBPNIPAM-Azo-OCH_3_ (*M*_n GPC_ = 8400 g·mol^−1^, *M*_w_/*M*_n_ = 1.82) aqueous solution (2 mg·mL^−1^) prepared under different time intervals in CHCl_3_ at room temperature with UV light of 365 nm ((**A**) *trans-cis* photoisomerization) and further visible light ((**B**) *cis-trans* photoisomerization) irradiation. First-order for *trans-cis* (**C**) and *cis-trans* (**D**) photoisomerization of HBPNIPAMs (HBPNIPAM-Azo-OC_3_H_7_: *M*_n GPC_ = 7300 g·mol^−1^, *M*_w_/*M*_n_ = 2.13; HBPNIPAM-Azo-OCH_3_; HBPNIPAM-Azo: *M*_n GPC_ = 8800 g·mol^−1^, *M*_w_/*M*_n_ = 1.78; HBPNIPAM-Azo-COOH: *M*_n GPC_ = 7400 g·mol^−1^, *M*_w_/*M*_n_ = 2.05).

Azobenzene chromophores and their derivatives (e.g., azobenzene-functionalized polymers) exhibit reversible *trans-cis-trans* isomerization behaviors, undergoing isomerization from *trans*- to *cis*-forms under UV irradiation (365 nm) and reverse transformation from *cis*- to *trans*- forms under vis irradiation [50,51,52,53]. Therefore, the *trans-cis* photoisomerization of BHPNIPAMs in aqueous solution (2 mg·mL^−1^) were investigated firstly at room temperature. Using HBPNIPAM-Azo-OCH_3_ as an example, UV–Vis spectroscopy was used to record the process of photoisomerization during irradiation of the samples with 365 nm UV light; the UV–Vis absorption changes are shown in Figure 5A. Before irradiation, the maximum absorption at about 350 nm was assigned to the characteristic π–π* transition of azobenzene (*trans*-form), and the weak absorption band at about 427 nm was attributed to the n–π* transition of azobenzene (*cis*-form). As shown in Figure 5A, the strong absorption at about 350 nm decreased rapidly, and the weak peak slightly increased with the irradiation time, which suggested that the *trans*- form of the azobenzene changed rapidly to the *cis*- form under UV irradiation. Along with irradiation, the *trans*- form of azobenzene did not completely disappear as there was still the presence of 36.5% *trans*- form of azobenzene in the HBPNIPAM-Azo-OCH_3_ [51]. A similar behavior was also observed with HBPNIPAM-Azo-OC_3_H_7_, HBPNIPAM-Azo, and HBPNIPAM-Azo-COOH polymers. The maximum absorbances corresponding to the *trans*- and *cis*- forms are listed in Table 2. When photo-stationary state was reached, the contents of the *trans* -form in HBPNIPAM-Azo-OCH_3_, HBPNIPAM-Azo-OC_3_H_7_, HBPNIPAM-Azo, and HBPNIPAM-Azo-COOH polymers were 38.4, 36.5, 33.6,and 40.0, respectively (Table 2). The rates of *trans-cis* photoisomerization were calculated from absorption changes of the *trans*- form. The kinetics of the *trans-cis* isomerizations of HBPNIPAMs is presented in Figure 5C. The first-order rate constants were determined by Equation (2):

(2) $$In\left(\frac{A_{\infty }-A_{0}}{A_{\infty }-A_{t}}\right)=-k_{e}t$$

where *A*_∞_, *A*_0_, and *A*_t_ are absorbance intensities of the *trans*- form after 365 nm UV irradiation at infinite time, zero and t seconds, respectively. The *trans-cis* isomerization rate constants (*k*_e_) of the four HBPNIPAMs were 0.1740, 0.2378, 0.0228, and 0.0450 s^−1^ (Table 2). The rate constants of HBPNIPAM-Azo-OC_3_H_7_ and HBPNIPAM-Azo-OCH_3_ were found to be much higher than those of HBPNIPAM-Azo and HBPNIPAM-Azo-COOH, but these results are quite different from those reported in the literature [54]. The reason for the higher rate constants in the former two polymers was apparently due to self-assembly of HBPNIPAM-Azo-OC_3_H_7_ and HBPNIPAM-Azo-OCH_3_ resulting in many more core-shell nanoparticles containing the ordered azobenzene cores in aqueous solution, which enhanced the speed of the response.

In addition, reverse *cis-trans* isomerization of the irradiated solutions of HBPNIPAMs were investigated under vis irradiation. As shown in Figure 5B, the absorption at 350 nm was rapidly restored to the initial state of HBPNIPAM-Azo-OCH_3_ within about 740 s and the absorption of *cis*-form at 427 nm was also restored reaching equilibrium between *cis*- and *trans*- forms *i.e.*, the same state as before UV irradiation. Therefore, the *cis-trans* photoisomerization kinetics of the HBPNIPAMs solution can be fitted to Equation (3) as follows:

(3) $$\mathrm{Ln}\left(\frac{A_{\infty }-A_{0}}{A_{\infty }-A_{t}}\right)=k_{H}t$$

where *A*_∞_, *A*_0_, and *A*_t_ are absorbance intensities at 350 nm at infinite time, zero, and *t* seconds, respectively. A good fit to the first order *cis-trans* isomerization is shown in Figure 5D. The *cis-trans* rate constants (*k*_H_) of HBPNIPAMs were 8.96 × 10^−3^, 4.96 × 10^−3^, 3.52 × 10^−3^, and 4.69 × 10^−3^ s^−1^ without significant difference among them (Table 2). These results confirmed that the *trans-cis-trans* isomerization of azobenzene in the HBPNIPAMs can be reversibly irradiated between the UV and Vis lights, and therefore they may have potential applications as light stimuli-responsive materials.

### 3.3. Self-Assembly of Hyperbranched Poly(N-isopropylacrylamide)s End-Capped with Different Azobenzene Chromophores (HBPNIPAM-Azo-OC_3_H_7_, HBPNIPAM-Azo-OCH_3_, HBPNIPAM-Azo, and HBPNIPAM-Azo-COOH)

**Figure 6:**
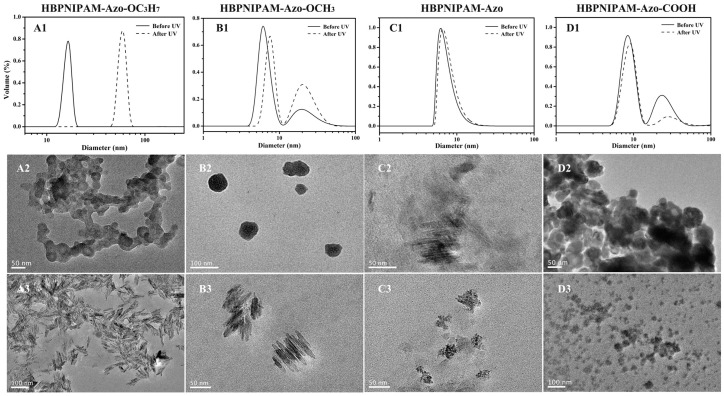
Intensity-weighted size distributions of hyperbranched poly(*N*-isopropylacrylamide)s solutions (2 mg·mL^−1^) including HBPNIPAM-Azo-OC_3_H_7_ (**A1**); HBPNIPAM-Azo-OCH_3_ (**B1**); HBPNIPAM-Azo (**C1**); and HBPNIPAM-Azo-COOH (**D1**) during UV irradiation. Transmission electron microscope (TEM) pictures of HBPNIPAMs solutions before (**A2**,**B2**,**C2**,**D2**) and after UV irradiation (**A3**,**B3**,**C3**,**D3**).

Previous work has shown that an amphiphilic hyperbranched polymer could self-assemble directly into nanoparticles in an aqueous solution [40]. In this study, we show that self-assembly leads to reversible micelles by light irradiation. Hyperbranched poly(*N*-isopropylacrylamide)s end-capped with azobenzene chromophores reversibly formed micelles in response to switching between UV and visible light irradiation [23,26,27,40], as confirmed by dynamic light scattering (DLS) analysis and TEM observations (Figure 6). Before UV irradiation, DLS measurements (Figure 6A1) indicated that HBPNIPAM-Azo-OC_3_H_7_ self-assembled to form normally distributed single micelles with an average size of about 17 nm, which were composed of multimolecular HBPNIPAM-Azo-OC_3_H_7_ chains with the hydrophobic core (terminated 4-propoxyazobenzene) and the hydrophilic shell (branched PNIPAM chain). After UV irradiation, there was an obvious increase in the average diameter to 58 nm but the size distribution remained unchanged, which indicated that the HBPNIPAM-Azo-OC_3_H_7_ subsequently self-assembled to generate the larger micelles. The hydrophobic *trans*- form of the azobenzene transformed into the strong polar *cis*- form under UV irradiation, and the tight cores of regular *trans*- form azobenzene with an ordered array transformed to the *cis*- form, which packed into the loose cores as the unordered array. Due to the exposed propoxy group from azobenzene, the loose cores of the *cis*-form azobenzene became an unstable state in forming the much larger micelles from the combination of the primary nanoparticles. TEM showed the overall shape of the particles (Figure 6). The results revealed that HBPNIPAM-Azo-OC_3_H_7_ could self-assemble into spherical aggregates in the range of 10–25 nm (Figure 6A2), which is in good agreement with DLS analysis. However, the spherical aggregates were unstable and gathered together to form bead-necklace type particles (Figure 6A2) when sample became dry on the copper mesh. Thus the spherical aggregates of HBPNIPAM-Azo-OC_3_H_7_ were sensitive to environmental stimulation. Most interestingly, the spherical micelles changed to nanorods (Figure 6A3), and the particle size increased to 30–80 nm during UV irradiation. These results clearly showed that UV light irradiation induced changes in the aggregate micelles of HBPNIPAM-Azo-OC_3_H_7_.

When 4-methoxyazobenzene was substituted in place of the 4-propoxyazobenzene at the end of HBPNIPAM to form HBPNIPAM-Azo-OCH_3_, DLS results (Figure 6B1) revealed the formation of micelles with much wider bimodal distribution, indicating the presence of two types of micelles with average sizes of about 5 and 21 nm. The two types of aggregates were composed of unimolecular and multimolecular HBPNIPAM-Azo-OCH_3_ chains, respectively. The relative content of the smaller aggregates was much higher than that of bigger aggregates (Figure 6B1). TEM imagery (Figure 6B2) also showed few spherical micelles with sizes in the range of 15–50 nm in diameter. Because the para substituent (hydrophobic-OC_3_H_7_) of azobenzene chromophore was changed into weakly hydrophobic-OCH_3_ in HBPNIPAM-Azo-OCH_3_, the aggregation ability of the multimolecular polymer became weaker. Therefore, lots of smaller unimolecular aggregates resulted in HBPNIPAM-Azo-OCH_3_ (Figure 6B1), while the bigger aggregates became scarcer. As for HBPNIPAM-Azo containing no para substituent with azobenzene, the hydrophobicity of azobenzene became much weaker, and therefore, only unimolecular aggregates (about 5 nm) formed in aqueous solution (Figure 6C1), which could not be seen in TEM images (Figure 6C2,C3). However, when the para substituent of azobenzene chromophore was carboxyl, which is strongly polar, the intensity-weighted size distribution of HBPNIPAM-Azo-COOH (Figure 6D1) solution was similar to that of HBPNIPAM-Azo-OCH_3_ (Figure 6B1). The HBPNIPAM-Azo-COOH showed few multimolecular (17 nm) but many unimolecular (8 nm) aggregates coexisting in aqueous solution (Figure 6D1), indicating that the aggregation ability of unimolecular aggregates was realized through hydrogen bonds from carboxyl groups in the tight azobenzene (*trans*- form) cores. The above results show that the hydrophobicity from the end-capped azobenzene structure had great influence on self-assembly behavior, including the size and components of aggregates.

After UV irradiation, the stimuli-responsive behavior of the differently modified HBPNIPAMs was also different. All the spherical aggregates of HBPNIPAM-Azo-OC_3_H_7_ changed to nanorods of larger size during UV irradiation, indicating that the unstable cores from *cis*- form azobenzene-OC_3_H_7_ were driven together to form nanorods due to the hydrophobic-OC_3_H_7_. When *cis*-form azobenzene-OCH_3_ was packed into the loose cores as an unordered array, after UV irradiation the spherical aggregates of HBPNIPAM-Azo-OCH_3_ also changed to stable nanorods^18^ of the same size, as shown in Figure 6B1,B3. In this case, some unstable unimolecular particles formed the bigger loose structure (7 nm), and partly assembled to form new nanorods with the same size. Although HBPNIPAM-Azo was affected by *cis*-form azobenzene in the loose cores, unimolecular aggregates of HBPNIPAM-Azo changed little, as shown in Figure 6C1,C3. As for HBPNIPAM-Azo-COOH shown in Figure 6D1,D3, the aggregation ability of the unimolecular aggregates weakened under UV irradiation. However, some unstable particles dissociated to form many more unimolecular aggregates, leading to a bigger loose structure (10 nm), which was probably due to competition of water molecules and carboxyl groups for hydrogen bonds in the loose Azo-COOH (*cis*-form) structure.

**Figure 7:**
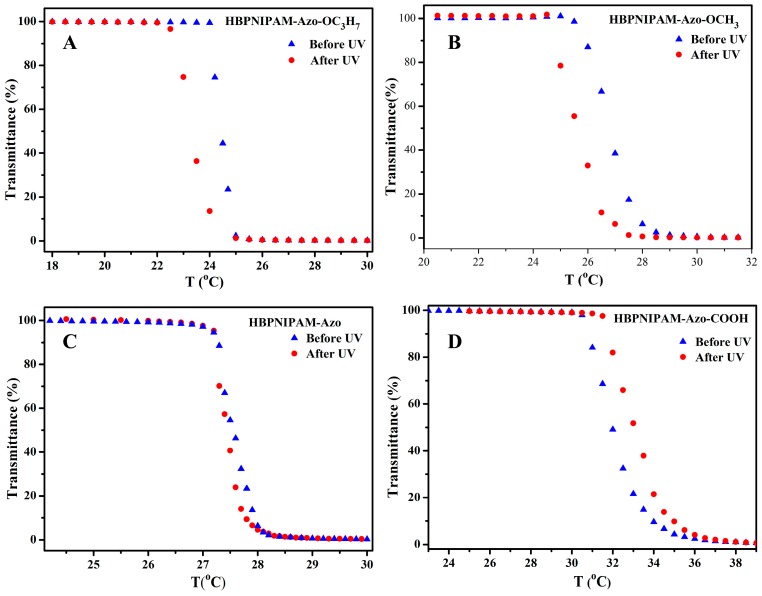
Temperature dependence of the transmittance at 550 nm light through hyperbranched poly(*N*-isopropylacrylamide)s (HBPNIPAM) aqueous solutions (2 mg·mL^−1^) during UV irradiation: HBPNIPAM-Azo-OC_3_H_7_ (**A**); HBPNIPAM-Azo-OCH_3_ (**B**); HBPNIPAM-Azo (**C**); and HBPNIPAM-Azo-COOH (**D**).

The above results showed that azobenzene structures at the end of the HBPNIPAM exerted great influence on self-assembly behavior, which in turn led to a change in LCST during UV irradiation. It is well known that PNIPAM is a thermo-responsive material, which shows reversible major phase-transition behavior at its lower critical solution temperature (LCST) of about 32 °C. The introduction of the azobenzene moiety into the PNIPAM chain resulted in significant changes of the LCST by undergoing reversible *trans-cis* photoisomerization and changes in geometry and polarity of azobenzene units upon UV irradiation [24,25,26]. On the basis of this valuable behavior of PNIPAM and azobenzene, here we studied the temperature dependence of the 550 nm optical transmittance to measure the changes of LCSTs of the BHPNIPAMs in aqueous solution (2 mg·mL^−1^) between UV and vis light irradiation; the results are presented in Figure 7. HBPNIPAM-Azo-OC_3_H_7_ solution remained almost transparent below 24 °C (Figure 7A), and there was a sharp decrease of transmittance above 24 °C, indicating phase separation without precipitation. The LCST of HBPNIPAM-Azo-OC_3_H_7_ at 24 °C was lower than the normal value (32 °C), because it was affected by the hydrophobicity of the azobenzene structure at the end of HBPNIPAM [4]. When the HBPNIPAM-Azo-OC_3_H_7_ solution was exposed to UV light until the *trans-cis* photoisomerization state was complete, the LCST value clearly decreased to about 22 °C, *i.e.*, the difference in temperature (ΔT) was −2 °C with HBPNIPAM-Azo-OC_3_H_7_ solution (as shown in Table 1), which was due to changes in aggregates during UV irradiation. The initially formed spherical micelles completely transformed to nanorods of larger size, which are more responsive to temperature [48]. Similarly, when the number of multimolecular aggregates in the HBPNIPAM-Azo-OCH_3_ solution increased significantly, there was a small decrease in the LCST value (24.5 °C) compared to that of the initial state (25.5 °C), *i.e.*, Δ*T* = −1 °C, as can be seen in Figure 7B. However, the LCST value of the HBPNIPAM-Azo solution (Figure 7C) remained almost constant during UV irradiation, because unimolecular aggregates of HBPNIPAM-Azo changed little or not at all. As for HBPNIPAM-Azo-COOH in Figure 7D, some unstable multimolecular particles dissociated to form many more unimolecular aggregates. Moreover, after UV irradiation, the *trans*- form of the azobenzene changed rapidly to the *cis*- form, and the polarity of the *cis*- form was higher than that of the *trans*- form azobenzene [24]. Therefore, HBPNIPAM-Azo-COOH with the higher polarity of the *cis*- form was much more hydrophilic, leading to a LCST increase from 30.5 to 31.5 °C.

**Figure 8:**
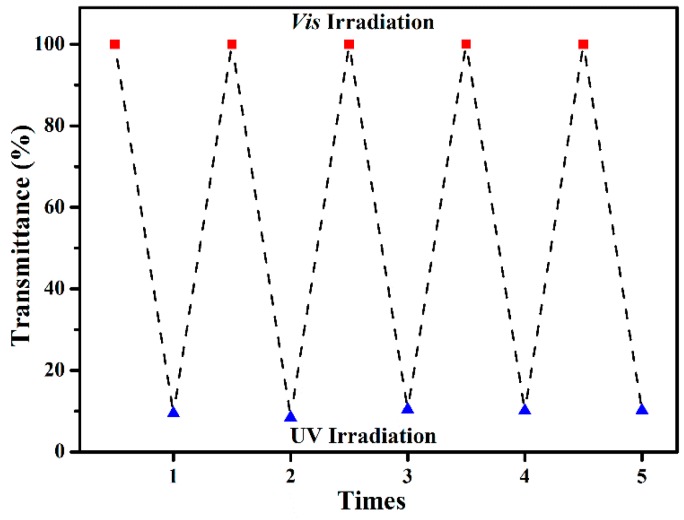
Reversible changes of transmittance at 550 nm for the HBPNIPAM-Azo-OC_3_H_7_ (*M*_n GPC_ = 7300 g·mol^−1^, *M*_w_/*M*_n_ = 2.13) aqueous solution (2 mg·mL^−1^) at 24 °C under alternating irradiation of UV and visible light.

Figure 8 shows the changes in transmittance of HBPNIPAM-Azo-OC_3_H_7_ solution (2 mg·mL^−1^) irradiated between UV and vis light at 24 °C. These results demonstrated that the transmittance between 99% and 10% was reversibly switched five times by the tuning of UV and vis light irradiation (Figure 8). Thus azobenzene chromophores exhibit reversible *trans-cis-trans* isomerization under alternating UV and vis light, and the phase transition changes of the HBPNIPAM with azobenzene end groups could be reversibly controlled by light (Figure 8).

## 4. Conclusions

A series of differently azobenzene-terminated hyperbranched poly(*N*-isopropylacrylamide)s, (HBPNIPAMs), *i.e.*, HBPNIPAM-Azo-OC_3_H_7_, HBPNIPAM-Azo-OCH_3_, HBPNIPAM-Azo, and HBPNIPAM-Azo-COOH, were designed and synthesized via atom transfer radical polymerization (ATRP) of *N*-isopropylacrylamide (NIPAM) using different functional initiators. All the HBPNIPAMs were similar in terms of their branching structure, content of azobenzene chromophores, and the absolute weight/average molecular weight (*M*_w MALLS_). HBPNIPAMs exhibited reversible *trans-cis-trans* isomerization behavior by switching between UV (365 nm) and vis irradiation. The *trans*-*cis* isomerization rate constant (*k*_e_) of HBPNIPAM-Azo-OC_3_H_7_ (0.1740 s^−1^) or HBPNIPAM-Azo-OCH_3_ (0.2378 s^−1^) was much higher than that of HBPNIPAM-Azo (0.0228 s^−1^) or HBPNIPAM-Azo-COOH (0.0450 s^−1^), which was due to self-assembly of HBPNIPAM-Azo-OC_3_H_7_ and HBPNIPAM-Azo-OCH_3_ by forming more or bigger core-shell nanoparticles in aqueous solution. UV irradiation led to changes in LCST values and changes of the aggregate micelles of HBPNIPAMs, relating to the end-capped azobenzene structure. These results showed that azobenzene structures at the end of the HBPNIPAM exerted great influence on their LCST and self-assembly behavior.

## Figures and Tables

**Table 1:** Characteristic data of linear poly(*N*-isopropylacrylamide) (LPNIPAM) and hyperbranched PNIPAMs (HBPNIPAMs).

Sample	Time ^c^ (h)	*Conv.*^d^ (%)	*M_n_*_GPC_^e^ (g·mol^−1^)	*M*_w_/*M_n_* ^e^	*M*_w MALLS_^f^ (g mol^−1^)	*α* ^g^	*g′* ^h^	LCST ^i^ °C	Δ*T ^j^*
LPNIPAM ^a^	60	94.3	4100	1.23	-	0.90	1.00	32.0	0
HBPNIPAM-Azo-OC_3_H_7_^b^	24	98.8	7300	2.13	65,200	0.26	0.60	24.0	−2.0
HBPNIPAM-Azo-OCH_3_^b^	45	97.0	8400	1.82	50,400	0.28	0.73	25.5	−1.0
HBPNIPAM-Azo ^b^	48	98.1	8800	1.78	57,900	0.23	0.65	27.0	0
HBPNIPAM-Azo-COOH ^b^	23	92.0	7400	2.05	52,500	0.25	0.59	30.5	1.0

^a^ Linear poly(*N*-isopropylacrylamide) (PNIPAM) was synthesized via atom transfer radical polymerization (ATRP) under the condition of [NIPAM]:[*t*-BB*i*B]:[CuCl]:[Me_6_TREN] = 30:1:1:3, and isopropyl alcohol/water (2:1, m/m) as mixed solvents at 25 °C; ^b^ Hyperbranched poly(*N*-isopropylacrylamide)s (HBPNIPAMs) were synthesized via ATRP under the condition of [NIPAM]_0_:[MBA]_0_:[initiator]_0_:[CuCl]_0_:[Me_6_TREN]_0_ = 30:0.9:1:1:3 and isopropyl alcohol/water (2:1, m/m) as mixed solvents at 25 °C. Initiators were C_3_H_7_O-Azo-Br, CH_3_O-Azo-Br, Azo-Br, and HOOC-Azo-Br; ^c^ Polymerization time; ^d^ Conversion of the NIPAM determined by an HP-689 gas chromatography. Isopropyl alcohol was used as the internal standard; ^e^ Determined by gel permeation chromatography (GPC) using tetrahydrofuran (THF) as the eluent. *M*_n_: the number-average molecular weight. *M*_w_/*M*_n_: molecular weight distribution; ^f^ The absolute weight/average molecular weight, measured by multi-angle laser light scattering (MALLS) detector; ^g^ The Mark–Houwink exponent, [η] = *KM*^α^; ^h^ The Zimm branching factor, *g*′ = [η]_branched polymer_ /[η]_linear polymer_; ^i^ Lower critical solution temperature (LCST) of polymer solution (2 mg·mL^−1^) in water was determined by optical transmittance of a 550 nm light *vs.* the temperature using an Agilent Cary 100; ^j^ LCST change value (ΔT) of PNIPAM solution after UV irradiation.

**Table 2:** Kinetic parameters for the *trans-cis-trans* isomerization of the hyperbranched poly(*N*-isopropylacrylamide)s (HBPNIPAMs).

Sample	λ_max_ (*trans*) ^a^ nm	λ_max_ (*cis*) ^b^ nm	*k*_e_^c^ × 10^2^ s^−1^	*k*_H_^d^ × 10^3^ s^−1^	[*trans*] ^e^ %
HBPNIPAM-Azo-OC_3_H_7_	344	431	17.40	8.96	38.4
HBPNIPAM-Azo-OCH_3_	350	427	23.78	4.96	36.5
HBPNIPAM-Azo	322	425	2.28	3.52	33.6
HBPNIPAM-Azo-COOH	329	425	4.50	4.69	40.0

^a^ The maximum absorbance intensity of *trans-*form azobenzene; ^b^ The maximum absorbance intensity of *cis*-form azobenzene; ^c^ Rate constant of *trans-cis* isomerization; ^d^ Rate constant of *cis-trans* isomerization; ^e^ *trans*-form contents when photo-stationary state was reached.

## Supplementary Materials

The following can be found at www.mdpi.com/2073-4360/8/5/183/s1: ^1^H-NMR spectra of Tris[2-(dimethylamino)ethyl]amine (Me_6_TREN), C_3_H_7_OC-Azo-Br, CH_3_O-Azo-Br, Azo-Br, and HOOC-Azo-Br are shown in Figure S1–S5, respectively.

## References

[1] Heskins M., Gillet J.E. (1968). Solution properties of poly(*N*-isopropylacrylamide). J. Macromol. Sci. Chem..

[2] Wycisk A., Döring A., Schneider M., Schönhoff M., Kuckling D. (2015). Synthesis of β-cyclodextrin-based star block copolymers with thermo-responsive behavior. Polymers.

[3] You Y.Z., Hong C.Y., Pan C.Y., Wang P.H. (2004). Synthesis of a dendritic core-shell nanostructure with a temperature-sensitive shell. Adv. Mater..

[4] Lutz J.F. (2008). Polymerization of oligo(ethylene glycol) (meth)acrylates: Toward new generations of smart biocompatible materials. J. Polym. Sci. A Polym. Chem..

[5] Kawano T., Niidome Y., Mori T., Katayama Y., Niidome T. (2009). PNIPAM gel-coated gold nanorods for targeted delivery responding to a near-infrared laser. Bioconj. Chem..

[6] You Y.Z., Kalebaila K.K., Brock S.L., Oupický D. (2008). Temperature-controlled uptake and release in PNIPAM-modified porous silica nanoparticles. Chem. Mater..

[7] Klaikherd A., Nagamani C., Thayumanavan S. (2009). Multi-stimuli sensitive amphiphilic block copolymer assemblies. J. Am. Chem. Soc..

[8] Pan Y.V., Wesley R.A., Luginbuhl R., Denton D.D., Ratner B.D. (2001). Plasma polymerized *N*-Isopropylacrylamide: Synthesis and characterization of a smart thermally responsive coating. Biomacromolecules.

[9] Luo C.H., Zuo F., Zheng Z.H., Cheng X., Ding X.B., Peng Y.X. (2008). Tunable smart surface of gold nanoparticles achieved by light-controlled molecular recognition effection. Macromol. Rapid Commun..

[10] Chen T., Fang Q., Zhong Q., Chen Y., Wang J. (2015). Synthesis and thermosensitive behavior of polyacrylamide copolymers and their applications in smart textiles. Polymers.

[11] Bradley C., Jalili N., Nett S.K., Chu L.Q., Forch R., Gutmann J.S., Berger R. (2009). Response characteristics of thermoresponsive polymers using nanomechanical cantilever sensors. Macromol. Chem. Phys..

[12] Etika K.C., Jochum F.D., Cox M.A., Schattling P., Theato P., Grunlan J.C. (2010). Nanotube friendly poly(*N*-isopropylacrylamide). Macromol. Rapid Commun..

[13] Guo Z., Zhang Y.W., Huang W., Zhou Y.F., Yan D.Y. (2008). Terminal modification with 1-adamantylamine to endow hyperbranched polyamidoamine with thermo-/pH-responsive properties. Macromol. Rapid Commun..

[14] Li G., Shi L., An Y., Zhang W., Ma R. (2006). Double-responsive core-shell-corona micelles from self-assembly of diblock copolymer of poly(*t*-butyl acrylate-*co*-acrylic acid)-*b*-poly(*N*-isopropylacrylamide). Polymer.

[15] Zhang W.D., Zhang W., Cheng Z.P., Zhou N.C., Zhu J., Zhang Z.B., Chen G.J., Zhu X.L. (2011). Synthesis and aggregation behaviors of nonlinear multiresponsive, multihydrophilic block copolymers. Macromolecules.

[16] Men Y.J., Drechsler M., Yuan J.Y. (2013). Double-stimuli-responsive spherical polymer brushes with a poly(ionic liquid) core and a thermoresponsive shell. Macromol. Rapid Commun..

[17] Florian D.J., Theato P. (2009). Temperature- and light-responsive polyacrylamides prepared by a double polymer analogous reaction of activated ester polymers. Macromolecules.

[18] Li Y.B., He Y.N., Tong X.L., Wang X.G. (2005). Photoinduced deformation of amphiphilic azo polymer colloidal spheres. J. Am. Chem. Soc..

[19] Zhao Y. (2012). Light-responsive block copolymer micelles. Macromolecules.

[20] Kungwatchakun D., Irie M. (1988). Photoresponsive polymers. Photocontrol of the phase separation temperature of aqueous solutions of poly-[*N*-isopropylacrylamide-*co*-*N*-(4-phenylazophenyl)acrylamide]. Makromol. Chem. Rapid Commun..

[21] He J., Tremblay L., Lacelle S., Zhao Y. (2014). How can photoisomerization of azobenzene induce a large cloud point temperature shift of PNIPAM?. Polym. Chem..

[22] Jochumab F.D., Theato P. (2013). Temperature- and light-responsive smart polymer materials. Chem. Soc. Rev..

[23] Feng Z., Lin L., Yan Z., Yu Y.L. (2010). Dual responsive block copolymer micelles functionalized by NIPAM and azobenzene. Macromol. Rapid Commum..

[24] Akiyama H., Tamaoki N. (2007). Synthesis and photoinduced phase transitions of poly(*N*-isopropylacrylamide) derivative functionalized with terminal azobenzene units. Macromolecules.

[25] Jochum F.D., Borg L.Z., Roth P.J., Theato P. (2009). Thermo- and light-responsive polymers containing photoswitchable azobenzene end groups. Macromolecules.

[26] Yu B., Jiang X.S., Wang R., Yin J. (2010). Multistimuli responsive polymer nanoparticles on the basis of the amphiphilic azobenzene-contained hyperbranched poly(ether amine) (hPEA-AZO). Macromolecules.

[27] Zhang J., Liu H.J., Yuan Y., Jiang S.Z., Yao Y.F., Chen Y. (2013). Thermo-, pH-, and light-responsive supramolecular complexes based on a thermoresponsive hyperbranched polymer. ACS Macro. Lett..

[28] Ciampolini M., Nardi N. (1966). Five-coordinated high-spin complexes of bivalent cobalt, nickel, and copper with tris(2-dimethylaminoethyl)amine. Inorg. Chem..

[29] Fréchet J.M. J., Gitsov I., Aoshima S., Leduc M.R., Grubbs R.B. (1995). Self-condensing vinyl polymerization: An approach to dendritic materials. Science.

[30] Benoit D., Chaplinski V., Braslau R., Hawker C.J. (1999). Development of a universal alkoxyamine for “Living” free radical polymerizations. J. Am. Chem. Soc..

[31] Zou P., Yang L.P., Pan C.Y. (2008). One-pot synthesis of linear-hyperbranched diblock copolymers via self-condensing vinyl polymerization and ring opening polymerization. J. Polym. Sci. A Polym. Chem..

[32] Li Y., Armes S.P. (2005). Synthesis and chemical degradation of branched vinyl polymers prepared via ATRP: Use of a cleavable disulfide-based branching agent. Macromolecules.

[33] Zhang C.B., Zhou Y., Liu Q., Li S.X., Perrier S., Zhao Y.L. (2011). Facile synthesis of hyperbranched and star-shaped polymers by RAFT polymerization based on a polymerizable trithiocarbonate. Macromolecules.

[34] Li F., Xue X.Q., Huang W.Y., Yang H.J., Jiang B.B., Zheng Y.L., Zhang D.L., Fang J.B., Chen J.H., Kong L.Z. (2014). Ultrafast preparation of branched poly(methyl acrylate) through single electron transfer living radical polymerization at room temperature. Polym. Eng. Sci..

[35] Yang H.J., Bai T., Xue X.Q., Huang W.Y., Chen J.H., Qian X.L., Zhang G.Z., Jiang B.B. (2015). A simple route to vinyl-functionalized hyperbranched polymers: Self-condensing anionic copolymerization of allyl methacrylate and hydroxyethyl methacrylate. Polymer.

[36] Xue X.Q., Yang J., Huang W.Y., Yang H.J., Jiang B.B. (2015). Synthesis of hyperbranched poly(ε-caprolactone) containing terminal azobenzene structure via combined ring-opening polymerization and “click” chemistry. Polymers.

[37] Xue X.Q., Wang Y.L., Huang W.Y., Yang H.J., Chen J.H., Fang J.B., Yang Y., Kong L.Z., Jiang B.B. (2015). New insight into the ATRP of monovinyl and divinyl monomers. Macromol. Chem. Phys..

[38] Yang H.J., Bai T., Xue X.Q., Huang W.Y., Chen J.H., Qian X.L., Zhang G.Z., Jiang B.B. (2015). A versatile strategy for synthesis of hyperbranched polymers with commercially available methacrylate inimer. RSC Adv..

[39] Jiang Q.M., Huang W.Y., Yang H.J., Xue X.Q., Jiang B.B., Zhang D.L., Fang J.B., Chen J.H., Yang Y. (2014). Radical emulsion polymerization with chain transfer monomer: An approach to branched vinyl polymers with high molecular weight and relatively narrow polydispersity. Polym. Chem..

[40] Xue X.Q., Li F., Huang W.Y., Yang H.J., Jiang B.B., Zheng Y.L., Zhang D.L., Fang J.B., Kong L.Z., Zhai G.Q. (2014). Quadrangular prism: A unique self-assembly from amphiphilic hyperbranched PMA-*b*-PAA. Macromol. Rapid. Commun..

[41] Min K., Gao H.F. (2012). New method to access hyperbranched polymers with uniform structure via one-pot polymerization of inimer in microemulsion. J. Am. Chem. Soc..

[42] Gong H.D., Huang W.Y., Zhang D.L., Gong F.H., Liu C.L., Yang Y., Chen J.H., Jiang B.B. (2008). Studies on the development of branching in ATRP of styrene and acrylonitrile in the presence of divinylbenzene. Polymer.

[43] Huang W.Y., Zheng Y.L., Jiang B.B., Zhang D.L., Chen J.H., Yang Y., Liu C.L., Zhai G.Q., Kong L.Z., Gong F.H. (2010). Studies on the atom transfer radical branching copolymerization of styrene and acrylonitrile with divinyl benzene as the branching agent. Macromol. Chem. Phys..

[44] Jiang L., Huang W.Y., Xue X.Q., Yang H.J., Jiang B.B., Zhang D.L., Fang J.B., Chen J.H., Yang Y., Zhai G.Q. (2012). Synthesis of hyperbranched and highly branched methacrylates by self-condensing group transfer copolymerization. Macromolecules.

[45] Huang W.Y., Liu C., Yang H.J., Xue X.Q., Jiang B.B., Zhang D.L., Kong L.Z., Zhang Y., Komarneni S. (2014). Facile synthesis of highly branched poly(acrylonitrile-*co*-vinyl acetate)s with low viscosity and high thermal stability via radical aqueous solution polymerization. Polym. Chem..

[46] Xia Y., Yin X.C., Burke N.A.D., Stö1ver H.D.H. (2005). Thermal response of narrow-disperse poly(*N*-isopropylacrylamide) prepared by atom transfer radical polymerization. Macromolecules.

[47] Matyjaszewski K., Shipp D.A., Wang J.L., Grimaud T., Patten T.E. (1998). Utilizing halide exchange to improve control of atom transfer radical polymerization. Macromolecules.

[48] Xue X.Q., Yang J., Huang W.Y., Yang H.J., Jiang B.B., Li F., Jiang Y. (2015). Dual thermo- and light-responsive nanorods from self-assembly of the 4-propoxyazobenzene-terminated poly(*N*-isopropylacrylamide) in aqueous solution. Polymer.

[49] Huang W.Y., Yang H.J., Xue X.Q., Jiang B.B., Chen J.H., Yang Y., Pu H.T., Liu Y., Zhang D.L., Kong L.Z. (2013). Polymerization behaviors and polymer branching structures in ATRP of monovinyl and divinyl monomers. Polym. Chem..

[50] Xue X.Q., Zhu J., Zhang Z.B., Zhou N.C., Tu Y.F., Zhu X.L. (2010). Soluble main-chain azobenzene polymers via thermal 1,3-dipolar cycloaddition: Preparation and photoresponsive behavior. Macromolecules.

[51] Xue X.Q., Zhu J., Zhang Z.B., Cheng Z.P., Tu Y.F., Zhu X.L. (2010). Synthesis and characterization of azobenzene-functionalized poly(styrene)-*b*-poly(vinyl acetate) via the combination of RAFT and “click” Chemistry. Polymer.

[52] Xue X.Q., Yang J., Huang W.Y., Yang H.J., Jiang B.B. (2015). Preparation and characterization of novel side-chain azobenzene polymers containing tetrazole group. React. Funct. Polym..

[53] Huang T.C., Chen Y.Y., Chu C.C., Hsiao V.K.S. (2015). Optothermal switching of cholesteric liquid crystals: A Study of azobenzene derivatives and laser wavelengths. Materials.

[54] Caruso U., Diana R., Fort A., Panunzi B., Roviello A. (2006). Synthesis of polymers containing second order NLO-active thiophene and thiazole based chromophores. Macromol. Symp..

