# Supplementary material for: Light and Temperature as Dual Stimuli Lead to Self-Assembly of Hyperbranched Azobenzene-Terminated Poly(N-isopropylacrylamide)
Source: Polymers (Basel). 2016 May 7;8(5):183. doi: 10.3390/polym8050183 (PMC6432090; doi:10.3390/polym8050183)
Supplement: Supplementary file 1 [file polymers-08-00183-s001.pdf]

Supplementary Materials: Light and Temperature as Dual Stimuli Lead to Self-Assembly of Hyperbranched Azobenzene-Terminated Poly(N-isopropylacrylamide)

Wenyan Huang, Jing Yang, Yunqing Xia, Xuezi Wang, Xiaoqiang Xue, Hongjun Yang, Guifang Wang, Bibiao Jiang, Fang Li and Sridhar Komarneni

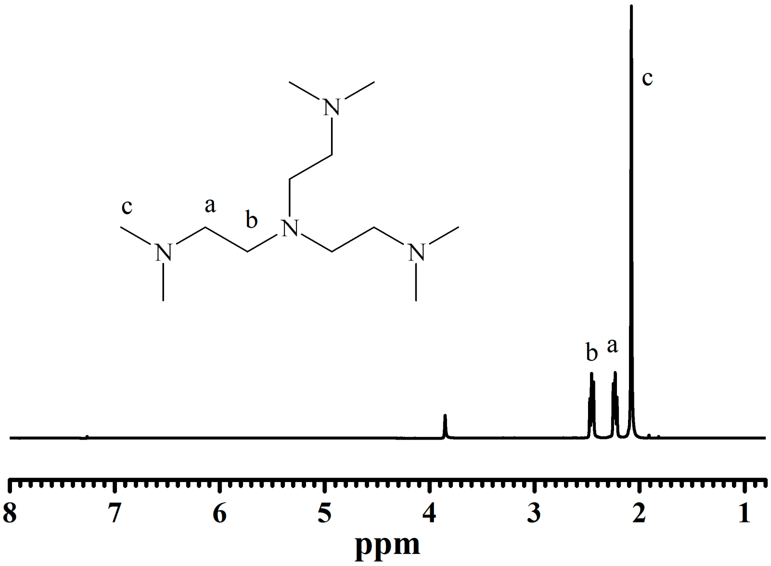

Fgure S1. ^{1}H-NMR spectrum of Tris[2-(dimethylamino)ethyl]amine (Me_6TREN) in $CDCl_3$.

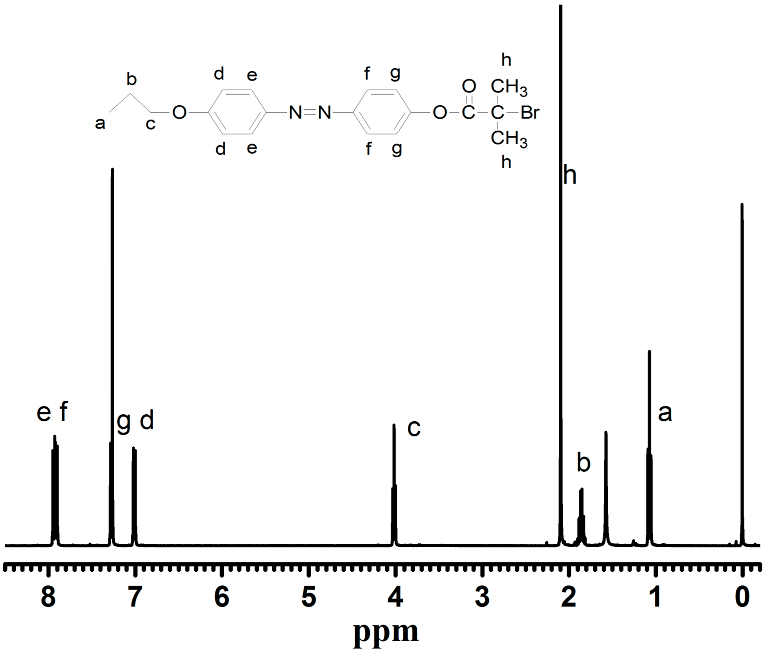

Figure S2. ^{1}H-NMR spectrum of C_3H_7OC-Azo-Br in $CDCl_3$.

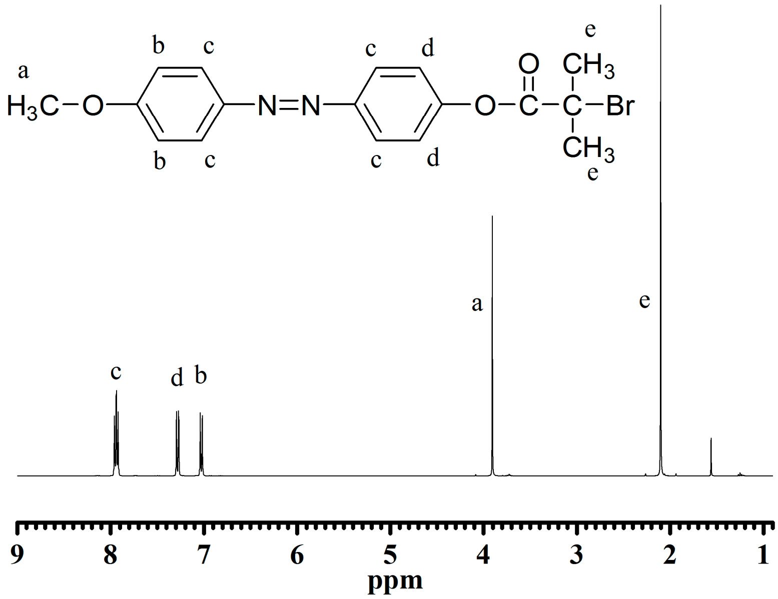

Figure S3. ^{1}H-NMR spectrum of CH_3O-Azo-Br in $CDCl_3$.

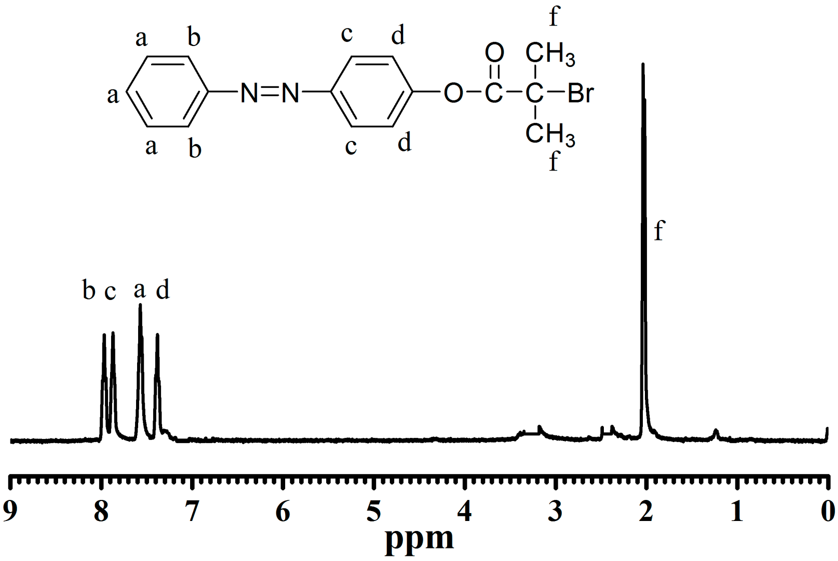

Figure S4. ^{1}H-NMR spectrum of Azo-Br in $CDCl_3$.

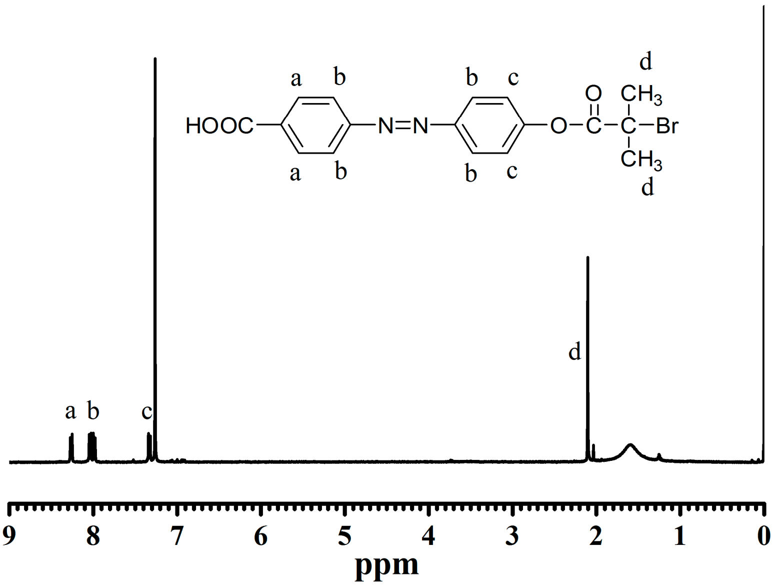

Figure S5. ^{1}H-NMR spectrum of HOOC-Azo-Br in $CDCl_3$.

© 2016 by the authors; licensee MDPI, Basel, Switzerland. This article is an open access article distributed under the terms and conditions of the Creative Commons Attribution (CC-BY) license (http://creativecommons.org/licenses/by/4.0/).
